# Atlanta—Returning Prosperity

**Published:** 1866-04

**Authors:** 


*Atlanta—Returning Prosperity.*—Since peace has returned to our land, Alanta, though by the ravages of war, almost utterly demolished, has, from the industrial energy of her people, attained, in some particulars, to her former importance and magnitude; and at the same rate of improvement which has been going on for twelve months past, business of every character will be greatly ahead of ante-war-times one year hence. While other departments of business are being pushed forward, medical matters are not neglected. Medical teaching has been re-established, and the prospects of Atlanta Medical College are far better for the ensuing Regular Summer Session, than the most sanguine could reasonably have anticipated, with the ruined condition of the country, and with the dilapidated condition of the college building, apparatus, &c. The improvements to the building, and the addition to the appliances necessary for instruction in the several departments of the Institution, are being made, and by the opening of the course in May, all will be complete as heretofore.

The Medical Profession of the city, as heretofore, have their regular Society meetings weekly, in which their Reports, discussions, consultations and weekly re-unions tend to cement the body medical and enhance their usefulness, individually, in the practice of their profession.

Druggists have accumulated—Phœnix-like, from the ashes of three or four drug houses seven or eight have risen. Some five or six establishments, as will be seen from our advertising pages, are in successful operation here. Taking into consideration the short time devoted to the preparation for business, it is a matter of astonishment that such general assortments and heavy stocks should be offered.

To physicians, druggists and builders finding it to their interest and convenience to make their purchases in Atlanta, we would say, their every want can be supplied. If, however, it is desirable to make purchases farther West, advertisements will be found in this Journal offering inducements to the trade.

